# Experiences of nursing students preparedness to migrate to online learning during COVID-19 lockdown in Namibia

**DOI:** 10.4102/hsag.v28i0.2427

**Published:** 2023-11-22

**Authors:** Daniel O. Ashipala, Pedro K. Mathias, Tadeus Shikukumwa

**Affiliations:** 1 Department of General Nursing Sciences, Faculty of Health Sciences and Veterinary Medicine, University of Namibia, Rundu, Namibia; 2 Distance Education Coordinator Learner Support services, Namibia College of Open Learning, Windhoek, Namibia

**Keywords:** COVID-19, experience, lockdown, migration, online learning, readiness

## Abstract

**Background:**

COVID-19 made it mandatory for Namibian education institutions to transition from traditional face-to-face classroom learning to online learning. Minimal time was available to prepare nursing students to adopt this model of learning, which subsequently influenced their learning experiences.

**Aim:**

The aim of the study was to explore and describe nursing students’ experiences regarding their preparedness to migrate to online learning during the COVID-19 lockdown at a public university in Namibia.

**Setting:**

Semi-structured interviews were conducted in English at the public university in Kavango East, Namibia.

**Methods:**

A qualitative approach utilising an exploratory and descriptive design was used. Convenience sampling and a semi-structured interview guide was used to assess the experiences of undergraduate nursing students. Data saturation was achieved after 15 interviews. ATLAS.ti 8 software assisted with management of data that was analysed inductively following the six steps of thematic analysis.

**Results:**

The following themes emerged from analysis of the data: (1) students’ readiness to migrate to online learning; (2) challenges faced by nursing students during the migration to online learning; and (3) strategies to support the transition from face-to-face to online learning.

**Conclusion:**

The study’s findings show that the student nurses were unprepared for online learning due to lack of skills and the ability to use technology to navigate online learning platforms. Access to online learning was also hampered by poor Internet connectivity and unreliable electronic devices.

**Contribution:**

These findings may be used to develop targeted interventions and strategies to mitigate challenges faced during transition from face-to-face to online learning.

## Introduction

The unexpected arrival of COVID-19 threw nursing education globally into disarray, necessitating a quick response from academic nursing institutions. The intense influence of the health crisis may have forever changed how future nurses will be educated and trained. It used to be that classroom instruction as well as any associated experiential learning or placements were done in-person (Oducado et al. 2019); however, the substantial turmoil caused by COVID-19 resulted in the education sector undergoing a paradigm shift, which resulted in a movement from face-to-face learning on a campus to remote learning in digital spaces, virtual environments and online platforms, that is, e-learning (Li & Lilani 2020). E-learning indicates that students are undergoing their learning online, rather than in-person (Mahmoud, El Magrabi & Mohamed 2015; Soriano & Oducado 2021; Voutilainen, Saaranen & Sormunen 2017).

In the time of a pandemic such as COVID-19, the employment of information technology is likely to accelerate, thus technology is critical for transforming learning and teaching (Raheem & Khan 2020). Now more than ever before, considerable attention is being paid to the blending of educational technology and e-learning in higher learning (Williams et al. 2011), including in the field of healthcare. Online learning provides improved access to resources that are only available on the Internet (Regmi & Jones 2020), where details like location and the time needed for in-person teaching can be circumvented (Rae, Murray & McKenzie 2010).

It is important to note that even before the pandemic, certain countries had been moving education of nursing students to online platforms. (Betihavas et al. 2016). Yet in Namibia, online teaching was not considered useful given the country’s developing status. This changed with the advent of COVID-19, when educational institutions around the world moved swiftly to undertake learning and teaching online.

While e-learning could change the educational landscape by expanding the number of educational opportunities and driving an increase in new pedagogical and instructive approaches, some argue that this abrupt shift may have resulted in poor educational activity outcomes and a negative experience for users (Li & Lilani 2020; Platt, Amber & Yu 2014).

As nursing students embraced online learning during the pandemic, the public university wanted to assess their students’ perceptions and experiences regarding their readiness for the migration. This was essential to assess whether the students considered technology in education to be a beneficial alternative to face-to-face teaching (Chow et al. 2014). Moreover, because the nursing students’ perceptions regarding their readiness for online learning had an effect on attaining the goal of learning itself, it was useful to assess the students’ experiences of it. Therefore, the purpose of this study was to explore and describe nursing students’ readiness for e-learning during the pandemic lockdown at the public university in Kavango East, Namibia.

## Research methodology and design

This study utilised a qualitative, exploratory, descriptive research design as it assisted the researchers to assess the thoughts and feelings of the research participants. Such a design explores how people make sense of their surroundings, experiences and understandings of a phenomenon (Green & Thurgood 2018).

## Population, sample and setting

This study was conducted in English at the public university in Kavango East, Namibia. The university offers degrees in commerce, education and nursing. The accessible population of this study consisted of undergraduate nursing students (third–fourth year) who were enrolled for a Bachelor of Nursing Science (Clinical) (Honours) degree at the School of Nursing in 2021. In all, 15 participants were selected using the convenience sampling method. The inclusion criteria for participants in this study were:

- full-time third and fourth year undergraduate nursing students who were enrolled for the Bachelor of Nursing Science (Clinical) (Honours) at the public university in Kavango East, Namibia in 2021
- willingness to participate in the study
- availability at the time the study was conducted.

## Data collection

Data were collected in September 2022 by the researcher, who led all the interviews under the guidance of his research supervisors. The interviewees were required to sign their consent to be involved in the study and it was explained that they could suspend their participation at any time with no penalties. Semi-structured interviews were held according to an interview guide. The interviews were conducted by the researcher at a location that was convenient for the participants and lasted between 40 and 50 min each. During the interviews, the researcher used an audio recorder and also took notes. Data saturation was reached after 15 interviews. This is the point in data collection when all important issues or insights are exhausted from data, which signifies that the conceptual categories that comprise the research are ‘saturated’ as the collection and/or analysis of additional data adds nothing new to a piece of research. As the interviews were held in person, precautionary measures were taken against COVID-19. Infection control measures included keeping a COVID-19 register, wearing masks, having adequate ventilation and adhering to social distancing. A pilot study was initially administered with three interviewees from the sampling unit, in order to determine the effectiveness and appropriateness of the questions, as well as to identify if the participants had any difficulties. Corrections were made accordingly following this pilot. As the student participants were selected according to the same benchmarks as the target population, the data gathered from these interviews were incorporated into the main study. The following research questions were posed:

- What were your experiences regarding your readiness to migrate to online learning during the COVID-19 lockdown at the public university in Kavango East, Namibia?
- What challenges did you face during the migration to online learning at the public university in Kavango East, Namibia?
- What strategies can be used to support the transition of nursing students from face-to-face (traditional learning) to online learning (e-learning) at the public university in Kavango East, Namibia?

### Data analysis

The data collected in qualitative research are non-numerical and are usually presented in the form of written material, video tapes, audiotapes, and photographs (Brink, Van der Walt & Van Rensburg 2018). In this qualitative study, the researcher followed an inductive approach using the thematic analysis technique to analyse the data. The data gleaned from the audio recordings were transcribed verbatim, after which they were analysed using qualitative thematic analysis (Braun & Clarke 2021) utilised Braun’s six-step method of data analysis. This included:

- becoming familiar with the data;
- generating initial codes;
- coding data following Tesch’s nine steps;
- determining and reporting themes;
- defining and naming themes; and
- interpreting the data (Braun & Clarke 2021).

### Measures to ensure trustworthiness

The trustworthiness of the study was ensured by using the criteria of Lincoln and Guba (1988), namely credibility, dependability, transferability, and confirmability. Credibility was safeguarded by the researcher remaining in the field until the data were considered sufficient for a thorough comprehension of the phenomenon under investigation. In addition, a data analysis mirrored the data gathered for an audit trail. Dependability was achieved through an audit trail, which is available upon request. Transferability was achieved by including a dense description, which includes a comprehensive description of the research methods used and the direct quotes of participants for illustration purposes. This was vital to ensure that the discoveries, conclusion and suggestions were supported and that there was consensus between the researcher’s interpretations and the facts (Brink et al. 2018). Finally, the transferability of this study was achieved by focussing on a confirmability audit, which was undertaken by an independent expert researcher.

### Ethical considerations

After approval from the University of Namibia’s Research Ethics Committee and the Ministry of Health and Social Services (reference number 17/3/3/PM), ethical clearance to gather data was received from the School of Nursing Ethics Committee (SoNEC) (reference number 18/2022). Anonymity was ensured as the participants were assigned codes instead of using names. In this way, the interviewees were guaranteed confidentiality as no information collected during the study could be linked to any one of them. The data are safely stored on a computer that is encrypted with a password only known to the researcher, and will be disposed of following the university’s policy.

### Findings

The participants in this study were undergraduate nursing students who were pursuing a Bachelor’s degree in Nursing Science (Clinical) (Honours) at the public university in Kavango East, Namibia. The participants were all under the age of 30 years and were third or fourth year nursing students.

### Participants’ demographic data

In all, 15 undergraduate nursing students took part in the study – 10 of these undergraduates were fourth year nursing students and the remaining five undergraduates were third year nursing students. Table 1 indicates the characteristics of the study participants.

**TABLE 1:** Characteristics of study participants.

Characteristics	Total number
**Age (in years)**	
18–22	3
23–27	12
**Gender**	
Male	6
Female	9
**Marital status**	
Single	15
Married	0
**Year of study**	
Third	5
Fourth	10

### Presentation and discussion

The following themes emerged from the data analysis:

- students’ readiness to migrate to online learning;
- students’ experiences of migrating to online learning; and
- strategies to support the transition from face-to-face learning to online learning.

Table 2 summarises the study’s findings in the form of themes, sub-themes, codes and participants’ comments. Detailed descriptions of the themes and categories in this study are given below.

**TABLE 2:** Summary of findings.

Themes	Sub-themes
Students’ readiness to migrate to online learning	Students’ readiness or unpreparedness Adaptation from traditional teaching and learning
Nursing students’ experiences of readiness to migrate to online learning	Positive experiences by nursing students Challenges faced by nursing students
Strategies to support the transition from face-to-face to online learning	University support for students and lecturers Improve internet connectivity Adapt to online teaching and learning

#### Theme 1: Students’ readiness to migrate to online learning

This theme is a description of the participants’ perceptions of nursing students’ readiness to migrate to online learning. The sub-themes that emerged from this theme were: (1) students’ readiness or unpreparedness; and (2) adaptation from traditional teaching and learning.

**Sub-theme 1: Student’s readiness or unpreparedness:** This sub-theme focussed on the participants’ perceptions regarding their readiness to migrate to online learning during the COVID-19 lockdown. All the interviewees stated that they were not ready for online learning as there was no time to prepare for the transition from traditional learning,

‘Okay, based on the readiness of student nurses, I think student nurses were not ready to migrate to online learning.’ (P1, Female, Fourth year)

‘It was something that happened very fast and we didn’t expect that this disease was going change everything. So, it happened fast and we did not have enough time to get prepared for that.’ (P2, Female, Fourth year)

The finding above corresponds with that of a study conducted by Pete, Coopasami and Knight (2017), which reported that while students’ psychological readiness for e-learning was high, they lacked the technological and equipment readiness. For this reason, although e-learning could be used in nursing education, technological and equipment readiness require attention before it can be implemented effectively in this institution. Similar studies conducted by Makhado et al. (2022) and O’Keefe and Kristin (2022) indicated that most student nurses were not prepared, nor were they given any orientation to enable them to work independently with the tools that had become critical for their academic success.

**Sub-theme 2: Adaptation from traditional teaching and learning:** This sub-theme focusses on the participants’ views regarding the adaptation of traditional teaching and learning. The interviewees stated that they were used to in-person education and the migration and transition to online learning was new to them,

‘Most of the nursing students were only used to the routine or traditional education, which was face-to-face learning.’ (P4, Male, Fourth year)

The above finding corresponds with that of Lindgren and McDaniel (2012), who indicated that the fully online provision of transnational programmes raises many concerns regarding the learning experience, particularly about the extent of feedback and guidance that can be provided to students. For instance, the demonstration of practical knowledge online is much more difficult than in traditional classes (Wynter et al. 2022). Cesa-Bianchi, Shalev-Shwartz and Shamir (2011) also concluded that the Internet does not lend itself to the learning of subjects that involve practical and higher analytical reasoning.

#### Theme 2: Nursing students’ experiences of readiness to migrate to online learning

Participants described their experiences in relation to online learning during the lockdown and their experiences were both negative and positive. However, from these descriptions, it has been identified that most of their experiences were negative with one positive experience. Two sub-themes emerged from the central theme: positive experiences of online learning during the lockdown; and negative experiences of online learning during the lockdown.

**Sub-theme 1: Positive experiences of nursing students:** Data revealed that a few participants viewed online teaching and learning as having a positive impact. They explained how the new mode of teaching and learning is exciting as they learn at their own pace, that there are no disturbances or noise during online classes, and it is easy for them to attend classes in their comfort zones and learn new things every day, including how to use the online platforms,

‘I had space on my own way I could just listen without anyone disturbing me or without anyone making noise while I’m listening to the classes.’ (P2, Female, Fourth year)

‘On the positive side, I can say now at least I’ve learned how to use this online platform.’ (P1, Female, Fourth year)

Additionally, the participants emphasised that online learning created an opportunity to improve their computer skills and gain new knowledge about learning management systems such as Moodle and Panopto, as well as how they transit from the traditional way of teaching to online learning. Furthermore, some participants reported that online learning was cost-effective,

‘It exposed me to a new way of learning a new way of doing things. And then it also inspired me and motivated me to facilitate the concept of self-learning more, conducting my own research on different topics therefore broadening and widening my knowledge on different things.’ (P11, Female, Fourth year)

The above findings align with those of Mei Yuan (2021), who reported that most of the students had a positive attitude towards the transition to online learning. This is supported by a study conducted by Nguyen (2015), which reported that e-learning has a positive impact on the academic achievements of students in terms of reducing costs, saving time and increasing the accessibility of education, while enhancing academic performance. It was affirmed by Makhado et al. (2022) that online learning was identified as a vehicle through which learners are empowered to participate at their own time and reinforce self-directed learning. Wallace et al. (2020) meanwhile showed that despite the educational challenges during COVID-19, skills and creativity in nursing students increased.

**Sub-theme 2: Challenges faced by nursing students:** This theme is a description of the challenges faced by the participants regarding the migration to online learning during the COVID-19 lockdown. The sub-themes that emerged exposed both positive and negative experiences.

A number of students clarified that a lack of access to the Internet was one of the greatest challenges of learning from home. Some students had poor Internet connectivity, while others did not have Internet access at all. Given the shift to online learning, many public university students in Kavango East, Namibia, returned home; however, connectivity in the rural villages is sometimes not good at all; therefore, they had to leave their houses and walk to an area with good network reception,

‘I will end up not attending that class because of the poor connection. This does not only apply to me, but to my colleagues’ and lecturers as well.’ (P2, Female, Fourth year)

‘Due to poor internet connectivity, it took long to load when you are logging in to attend online and during the process you are missing a lot of the content.’ (P5, Male, Fourth year)

The above findings corroborate those of Alsoud and Harasis (2021), who revealed that it was primarily students from remote and disadvantaged areas who faced enormous challenges, such as a lack of accessibility to technology and the Internet. Studies conducted by Hasan and Khan (2020) and Azlan et al. (2020) reported mixed feelings about online learning, in that the flexibility of e-learning was popular, yet there were problems with poor network connectivity and limited data.

The participants said that they often had problems with their devices not being compatible with the online learning programmes; therefore, they needed to share devices with other students. This was particularly true of students with financial limitations,

‘It was challenging when it came to devices that we were using to attend on the online classes; sometimes the batteries are weak for phones and sometimes there is no power to charge phones and some students did not have gadgets.’ (P5, Male, Fourth year)

‘During the time when my laptop and cell phone were stolen, I had to attend class with a classmate of mine by sharing her cell phone.’ (P1, Female, Fourth year)

These findings correspond with a study conducted by Perera and Abeysekera (2022) during the COVID-19 outbreak, which found that 65.6% of students had access to technology through mobile phones, while 53.4% had engaged in e-learning for the first time. This was mainly because of the restrictions that were imposed during the COVID-19 pandemic. In addition, a study conducted by Ndibalema (2022) reported that nearly 56 million students worldwide were unable to use portable devices because of a lack of mobile connectivity.

The participants reported that online learning was introduced unexpectedly and was thus a new experience. They described a lot of apps and programmes, such as Moodle and Zoom, that they had never used before. Several students also lacked the information and communications technology (ICT) skills required to operate these platforms. They pointed out that some lecturers were inexperienced in using ICT themselves, which also delayed or slowed down the learning process,

‘Online learning was something new to me and it was challenging because I didn’t know where exactly to go, or how to manoeuvre around this online learning platform or this site.’ (P1, Female, Fourth year)

‘We were not orientated or induced to online education to at least be shown what to expect or how to go about online learning platforms.’ (P3, Female, Fourth year)

These findings correspond with those of a study conducted by Gautam (2022), which reported that a lack of ICT training, recursive electric power-cut, unavailability, poor bandwidth, and instability of the Internet were identified as the prime issues to be addressed by stakeholders. Similarly, Shindjabuluka, Ashipala and Likando (2022) reported in their study that nurse educators lack training on how to teach online. Furthermore, Mukasa et al. (2021), Rana, Garbuja and Rai (2021) and Serhan (2020) reported that students lacked the necessary experience to study online.

The challenges they encountered included poor Internet connectivity, problems with the teaching platforms and communication difficulties. Other studies conducted by Makhado et al. (2022) and Jaradat et al. (2021) revealed that clinical teaching and learning are difficult to accomplish online, leaving students without the necessary clinical experience.

#### Theme 3: Strategies to support the transition from face-to-face to e-learning

This theme is a description of what the interviewees said when they were asked to recommend future actions that might support the transition of nursing students from face-to-face to online learning at the public university in Kavango East, Namibia. The sub-themes in this theme are as follows: (1) university support; (2) connectivity security; and (3) adaptation to teaching and learning.

**Sub-theme 1: University support for students and lecturers:** The interviewees suggested that the University could benchmark the available user-friendly software. It was argued that this would help all the students, especially those who are not computer literate. They also mentioned that the University should provide pocket Wi-Fi to students so that they have access to an Internet connection,

‘I will recommend the management to look into possible ways on how they can maybe offer or distribute gadgets to those who are unable to afford the necessary gadgets that are needed to attend online classes.’ (P11, Female, Fourth year)

‘All I can say maybe in general terms is that students themselves should be informed in advance so that they can be physically, mentally, spiritually and psychologically prepared to be ready to interact in the gaze at their strengths and all the devices required and they have picked a suitable environment for them to conduct this transition.’ (P7, Male, Third year)

**Sub-theme 2: Connectivity security:** The participants mentioned that for online classes to be successful, there is a need for students to secure access to a strong Wi-FI network. They also noted the importance of saving mobile data for academic work and having back-up gadgets to stay connected to the internet,

‘I would try keeping my data because it was challenging, using the same phone for academic stuff and using the same data that I needed for social media.’ (P5, Male, Fourth year)

‘I would look for a back-up gadget in case the other one goes off or fail to connect to the learning platforms to still be able to connect to the class again.’ (P11, Female, Fourth year)

**Sub-theme 3: Adaptation to online teaching and learning:** The respondents indicated that online teaching and learning at the public university, can be enhanced if the university provides an orientation to students on how to use the relevant online platforms. They added that students should be divided into smaller groups for practical attendance and clinical placements to ensure enhanced practical learning opportunities. Finally, they suggested that the university should provide training on preventative measures for COVID-19 when students are in clinical areas,

‘I will say the University can have an orientation or an induction for nursing students and they are teaching them how to go about accessing their modules and attending online classes.’ (P9, Male, Fourth year)

‘I think the institution should use blended learning, using both traditional learning and online learning gives you an option whereby some of the parts of the modules you attend online so that people get exposed to the online learning platforms.’ (P10, Male, Fourth year)

These comments concur with those from a study conducted by Makhado et al. (2022), which reported that the introduction of a new learning modality should be accompanied by a thorough orientation of both learners and lecturers to facilitate effective learning. Lecturers must also develop innovative teaching methods and expose nursing students to clinical learning to acquire psychomotor skills and enhance their professional conduct (Shindjabuluka et al. 2022).

A study by Makgahlela et al. (2021) noted that institutions of higher learning need to put contingency plans in place for efficient communication in times of crises, while investing in efficient ICT infrastructure for remote learning, teaching and research. It was reported in a study by Agu et al. (2021) that it is important to have a full simulation in a nursing programme to mitigate the effect of similar future occurrences in the clinical practicum aspect of the curriculum.

## Strengths, limitations, and areas for further research

The strength of this study lies in the fact that the nursing students’ views were considered from their own perspectives. The study provided a broader understanding of, and insight into, the perceptions and experiences of undergraduate nursing students regarding their readiness to migrate to e-learning during the pandemic lockdown at the university. The use of an explorative design enabled the participants to freely narrate and interpret their perceptions and experiences, as well as provide recommendations for improvements. Because the study was only conducted at one campus, the findings are limited and cannot be generalised to other campuses or universities. Areas for potential future studies include the relationship between technology applications and learning outcomes.

## Conclusion

The purpose of the study was to explore and describe nursing students’ perceptions regarding their readiness to migrate to online learning during the COVID-19 lockdown. The study’s findings showed that the student nurses were not prepared to migrate to online learning, because of insufficient knowledge and skills concerning how to use Internet devices to navigate online learning platforms. The students’ access to online learning was also hampered by poor Internet connectivity and a lack of reliable electronic devices. These findings may be used to develop targeted interventions and strategies to mitigate challenges faced by students when it comes to online learning.

## References

[CIT0001] Agu, C.F., Stewart, J., McFarlane-Stewart, N. & Rae, T., 2021, ‘COVID-19 pandemic effects on nursing education: Looking through the lens of a developing country’, *International Nursing Review* 68(2), 153–158. 10.1111/inr.12663 33513283 PMC8014519

[CIT0002] Alsoud, A.R. & Harasis, A.A., 2021, ‘The impact of COVID-19 pandemic on student’s e-learning experience in Jordan’, *Journal of Theoretical and Applied Electronic Commerce Research* 16(5), 1404–1414. 10.3390/jtaer16050079

[CIT0003] Azlan, C.A., Wong, J.H.D., Tan, L.K., Huri, M.S.N.A., Ung, N.M., Pallath, V. et al., 2020, ‘Teaching and learning of postgraduate medical physics using Internet-based e-learning during the COVID-19 pandemic – A case study from Malaysia’, *Physica Medica* 80, 10–16. 10.1016/j.ejmp.2020.10.002 33070007 PMC7539931

[CIT0004] Betihavas, V., Bridgman, H., Kornhaber, R. & Cross, M., 2016, ‘The evidence for “flipping out”: A systematic review of the flipped classroom in nursing education’, *Nurse Education Today* 38(1), 15–21. 10.1016/j.nedt.2015.12.010 26804940

[CIT0005] Braun, V. & Clarke, V., 2021, ‘To saturate or not to saturate? Questioning data saturation as a useful concept for thematic analysis and sample-size rationales’, *Qualitative Research in Sport, Exercise and Health* 13, 201–216. 10.1080/2159676X.2019.1704846

[CIT0006] Brink, H., Van Der Walt, C. & Van Rensburg, G., 2018, *Fundamentals of research methodology for healthcare professionals*, 4th edn., Juta, Cape Town.

[CIT0007] Cesa-Bianchi, N., Shalev-Shwartz, S. & Shamir, O., 2011, ‘Online learning of noisy data’, *IEEE Transactions on Information Theory* 57(12), 7907–7931. 10.1109/TIT.2011.2164053

[CIT0008] Chow, S.K.Y., Wong, L.T., Chan, Y.K. & Chung, T.Y., 2014, ‘The impact and importance of clinical learning experience in supporting nursing students in end-of-life care: Cluster analysis’, *Nurse Education in Practice* 14(5), 532–537. 10.1016/j.nepr.2014.05.006 24916407

[CIT0009] Gautam, P., 2022, ‘Online education as perceived by teachers and students’, *Prithvi Journal of Research and Innovation* 4(1), 88–102. 10.3126/pjri.v4i1.50162

[CIT0010] Green, J. & Thurgood, N., 2018, *Qualitative methods for health research*, 4th edn., Sage, London.

[CIT0011] Hasan, N. & Khan, N.H., 2020, ‘Online teaching-learning during COVID-19 pandemic: Students’ perspective’, *The Online Journal of Distance Education and e-Learning* 8(4), 202–213.

[CIT0012] Jaradat, S. & Ajlouni, A., 2021, ‘Undergraduates’ perspectives and challenges of online learning during the Covid-19 pandemic: A case from the University of Jordan’, *Journal of Social Studies Education Research* 12(1), 149–173.

[CIT0013] Li, C. & Lilani, F., 2020, *The rise of online learning during the COVID-19 pandemic*, World Economic Forum, viewed 20 April 2020, from https://www.weforum.org/agenda/2020/04/coronaviruseducation-global-covid19-online-digital-learning/.

[CIT0014] Lincoln, Y.S. & Guba, E.G., 1988, Criteria for assessing naturalistic inquiries as reports, Sage, Beverly Hills, CA.

[CIT0015] Lindgren, R. & McDaniel, R., 2012, ‘Transforming online learning through narrative and student agency’, *Educational Technology & Society* 15(4), 344.

[CIT0016] Mahmoud, S.R., El Magrabi, N.M. & Mohamed, F.R., 2015, ‘Faculty of nursing teaching staff members and students’ attitudes toward e-learning’, *IOSR Journal of Nursing and Health Science* 4(4), 36–45.

[CIT0017] Makgahlela, M., Mothiba, T., Mphekgwana, P.M., Makhado, L., Selepe, M. & Mokwena, J.P., 2021, ‘Strategies to improve historically disadvantaged university staff’s wellbeing and administration of academic programmes during COVID-19: A descriptive survey study’, *South African Journal of Higher Education* 35(5), 125–137. 10.20853/35-5-4273

[CIT0018] Makhado, L., Musekwa, O.P., Luvhengo, M., Murwira, T., Lebese, R.T., Mulaudzi, M.T. et al., 2022, ‘An exploratory-descriptive study on the impact of COVID-19 on teaching and learning: The experiences of student nurses in the rural-based historically disadvantaged University of South Africa’, *INQUIRY: The Journal of Health Care Organization, Provision, and Financing* 59, 00469580221093191. 10.1177/00469580221093191 PMC9073114 35506683

[CIT0019] Mei Yuan, L., 2021, ‘Student’s attitude and satisfaction towards transformative learning: A research study on emergency remote learning in tertiary education’, *Creative Education* 12(1), 494–528. 10.4236/ce.2021.123035

[CIT0020] Mukasa, J., Otim, M., Monaco, B., Al Marzouqi, A., Breitener, P. & Jawahar, L., 2021, ‘Nursing students’ perspectives and readiness to transition to e-learning during COVID-19 in the UAE: A cross-sectional study’, *Advances in Medical Education and Practice* 12, 1505. 10.2147/AMEP.S335578 35221744 PMC8866987

[CIT0021] Ndibalema, P., 2022, ‘Constraints of transition to online distance learning in higher education institutions during COVID-19 in developing countries: A systematic review’, *E-Learning and Digital Media* 19(6), 595–618. 10.1177/20427530221107510

[CIT0022] Nguyen, T., 2015, ‘The effectiveness of online learning: Beyond no significant difference and future horizons’, *MERLOT Journal of Online Learning and Teaching* 11, 309–319.

[CIT0023] Oducado, R.M., Amboy, M.K.Q., Penuela, A.C. & Belo-Delariarte, R.G., 2019, ‘Correlation between theoretical classroom instruction and related learning experiences: Evidence from a Philippine Nursing University’, *International Journal of Scientific & Technology Research* 8(12), 3666–3670.

[CIT0024] Oducado, R.M.F., & Soriano, G.P., 2021. ‘Shifting the education paradigm amid the COVID-19 pandemic: Nursing students’ attitude to E learning’, *Africa Journal of Nursing and Midwifery* 23(1), 1–14.

[CIT0025] O’Keefe, R. & Auffermann, K., 2022, ‘Exploring the effect of COVID-19 on graduate nursing education’, *Academic Medicine* 97(3), S61. 10.1097/ACM.0000000000004537 34817402 PMC8855768

[CIT0026] Perera, R.H.A.T. & Abeysekera, N., 2022, ‘Factors affecting learners’ perception of e-learning during the COVID-19 pandemic’, *Asian Association of Open Universities Journal* 17(1), 84–100. 10.1108/AAOUJ-10-2021-0124

[CIT0027] Pete, M., Coopasami, M. & Knight, S., 2017, ‘E-learning readiness amongst nursing students at the Durban University of Technology’, *Health SA Gesondheid* 22(1), 300–306. 10.1016/j.hsag.2017.04.003

[CIT0028] Platt, C.A., Amber, N.W. & Yu, N., 2014, ‘Virtually the same?: Student perceptions of the equivalence of online classes to face-to-face classes’, *Journal of Online Learning and Teaching* 10(3), 489.

[CIT0029] Rae, H., Murray, G. & McKenzie, K., 2010, ‘Teachers’ attitudes to mainstream schooling’, *Learning Disability Practice* 13(10), 12–17. 10.7748/ldp2010.12.13.10.12.c8138

[CIT0030] Raheem, B.R. & Khan, M.A., 2020, ‘The significant use of ICT tools in English language teaching and learning with special reference to the COVID-19 pandemic’, *International Journal of Science, Engineering and Management* 5(10), 251–256.

[CIT0031] Rana, S., Garbuja, C.K. & Rai, G., 2021, ‘Nursing students’ perception of online learning amidst COVID-19 pandemic’, *Journal of Lumbini Medical College* 9(1), 6.

[CIT0032] Regmi, K. & Jones, L., 2020, ‘A systematic review of the factors–enablers and barriers–affecting e-learning in health sciences education’, *BMC Medical Education* 20(1), 1–18. 10.1186/s12909-020-02007-6 PMC7106784 32228560

[CIT0033] Serhan, D., 2020, ‘Transitioning from face-to-face to remote learning: Students’ attitudes and perceptions of using Zoom during COVID-19 pandemic’, *International Journal of Technology in Education and Science* 4(4), 335–342. 10.46328/ijtes.v4i4.148

[CIT0034] Shindjabuluka, R.N., Ashipala, D.O. & Likando, G.N., 2022, ‘COVID-19 as an enabler for enhancing online learning and teaching skills for nurse educators at the University of Namibia: Prospects and challenges’, *Health SA Gesondheid* 27, a1727. 10.4102/hsag.v27i0.1727 PMC8905325 35281284

[CIT0035] Voutilainen, A., Saaranen, T. & Sormunen, M., 2017, ‘Conventional vs. e-learning in nursing education: A systematic review and meta-analysis’, *Nurse Education Today* 50, 97–103. 10.1016/j.nedt.2016.12.020 28038371

[CIT0036] Wallace, C.L., Wladkowski, S.P., Gibson, A. & White, P., 2020, ‘Grief during the COVID-19 pandemic: Considerations for palliative care providers’, *Journal of Pain and Symptom Management* 60(1), e70–e76. 10.1016/j.jpainsymman.2020.04.012 PMC7153515 32298748

[CIT0037] Williams, B., Boyle, M., Molloy, A., Brightwell, R., Munro, G. & Brown, T., 2011, ‘Undergraduate paramedic students’ attitudes to e-learning: Findings from five university programs’, *Research in Learning Technology* 19(2), 89–100. 10.3402/rlt.v19i2.10311

[CIT0038] Wynter, K., Holton, S., Considine, J., Hutchinson, A.M., Munt, R., Williams, R. et al., 2022, ‘The impact of the COVID-19 pandemic on Australian hospital-based nursing and midwifery educators’, *Collegian* 29(3), 271–280. 10.1016/j.colegn.2021.10.007 34744480 PMC8556584

